# Applying to Fellowship During a Pandemic: Lessons Learned From the 2020-2021 Orthopaedic Spine Fellowship Application Cycle

**DOI:** 10.7759/cureus.22630

**Published:** 2022-02-26

**Authors:** Meghana Jami, Amy L Xu, Bo Zhang, Wesley M Durand, Farah N Musharbash, Jacob M Babu

**Affiliations:** 1 Department of Orthopedic Surgery, Johns Hopkins University, Baltimore, USA; 2 Spine Surgery, Illinois Bone & Joint Institute, Libertyville, USA

**Keywords:** medical education, orthopaedic surgery, fellowship, spine surgery, covid-19

## Abstract

Introduction

The COVID-19 pandemic resulted in a transition to a virtual format for all medical residency and fellowship application processes. Previous studies have discussed the successful implementation of virtual interviews, but a deep analysis of how the application process has changed for orthopedic surgery fellowship programs during the pandemic is lacking. The purpose of this study was to assess how COVID-19 impacted the orthopedic spine fellowship application and selection process.

Methods

A web-based survey was administered to the program directors of all 75 U.S. orthopedic surgery spine fellowship programs, which often can accept both orthopedic surgery and neurosurgery trained graduates. Questions focused on the changes from the 2019-2020 application cycle to the 2020-2021 cycle. We collected data on connecting with potential applicants, the general application process, and interviews offered by programs. Univariate analyses were used to compare data from the 2020-2021 cycle with the prior 2019-2020 cycle.

Results

Twenty-five of the 75 contacted program directors responded to our survey (33% response rate). The percentage of programs that offered virtual open houses/meet-and-greets increased from 20% in 2019-2020 to 52% in 2020-2021 (p=0.018). Social media use was unchanged (0.0% vs. 4.0%, p>0.05). Compared to the prior year, the number of interviews offered by programs increased by 1.5 (32.7 vs. 21.9 interviews, p=0.024). There were no significant differences in the numbers of applications received by programs, interview dates available, or separate interviews each candidate completed during an interview day (p>0.05 for all). The in-person interview was the most important factor in 2019-2020 for selecting applicants, whereas the virtual interview, letters of recommendation (LOR), and research were equally ranked as the most important factors in 2020-2021. Regarding interviews, 50% of respondents would “likely” consider virtual interviews as an option in addition to in-person interviews in the future, but most (55%) answered that it was “unlikely” that virtual interviews would entirely replace in-person interviews.

Conclusion

Spine fellowship programs were more likely to use virtual social events to recruit potential applicants, send out more interview invitations, and equally consider LOR and research with interview performance during an entirely virtual application cycle. Half of the program directors would consider offering virtual interviews as an option for future application cycles, which may help reduce costs associated with the process.

## Introduction

The COVID-19 pandemic has resulted in unprecedented changes to graduate medical education at all levels. Learning through in-person clinical care has been largely supplemented with virtual conferences, telemedicine, and independent time for other professional opportunities, such as research [1,2]. These changes have considerably disrupted the clinical experience of trainees in surgical specialties, which have seen a drastic reduction in case volume from the limitations on elective surgeries [3]. For orthopedic surgery residents, matching into fellowship is another important concern. For those intent on practicing spine surgery, fellowship training is essentially necessary for a career in the field, but only a limited number of coveted positions are available [4,5]. With the transition to a virtual format, the 2020-2021 fellowship application process also saw structural changes that may have implications for future cycles.

Previous studies have discussed how the shift to virtual interviews has been successfully implemented by fellowship programs for various surgical subspecialties during COVID-19 [6-9]. Virtual interviews offer the benefits of reduced costs and improved efficiency [10,11], but how these compare to traditional in-person interviews in regard to more personal characteristics, particularly the fit of a program and how well applicants can represent themselves over a screen, remains a concern for programs and applicants alike [6,9]. These concerns may lead to changes in practice regarding both fellowship program and applicant behaviors and selection during the application process. To our knowledge, no current literature assesses how such nuanced features of the application process have changed for orthopedic fellowships in the face of COVID-19. The purpose of this study was to determine how COVID-19 influenced the orthopedic spine fellowship application and selection process. To this end, we compared characteristics of the 2019-2020 and 2020-2021 application cycles.

This research was previously presented as a podium presentation at the 2021 Orthopaedic Research and Education Foundation Northeast Regional Symposium.

## Materials and methods

Survey design and administration

A survey was administered to all 75 orthopedic spine fellowship programs participating in the San Francisco Matching Program (SF Match) using web-based software (surveymonkey.com, Palo Alto, CA). Depending on the contact information publicly available for a given program, surveys were e-mailed to fellowship program directors or program coordinators with instructions for the program director to complete the survey. In total, the program directors of 25 out of 75 spine fellowship programs completed the survey (33.3% response rate). The survey consisted of 19 questions across three sections: (1) Connecting with potential applicants, (2) general application process, and (3) interviews. The survey was designed to detect changes in these categories from the 2019-2020 application cycle to the 2020-2021 cycle. Questions were worded to elicit responses from the perspective of the spine fellowship program rather than from the perspective of the applicant. This study was deemed exempt from institutional review board review by the study institution.

Statistical analysis

Univariate statistics were performed to analyze individual questions. To analyze changes between the 2019-2020 and 2020-2021 application cycles, bivariate analyses were performed using student's t-tests for parametric continuous variables, Mann-Whitney U tests for non-parametric continuous variables, and Chi-Squared tests for categorical variables. We used an "average ranking" algorithm to analyze the rank-choice questions, which assessed the importance of various factors in the applicant selection process for a given year. This algorithm was based on the following equation: (x1w1 + x2w2+....+xnwn)/(total response count), where x represents the response count for each answer choice and w represents the weight of the ranked position. This "average ranking" equation has been previously validated for interpreting rank-choice survey questions [12]. Statistical significance was set at p<0.05 for all statistical tests. All statistical analyses were performed using STATA version 15.0 (StataCorp LLC, College Station, Texas).

## Results

Questions on connecting with potential applicants

Communication via e-mail was the most common method used to inform potential applicants about a given program in both the 2019-2020 (56%) and 2020-2021 application cycles (68%) (Table 1). Compared to the prior year, the percentage of programs which offered virtual open houses/meet-and-greets increased from 20% to 52% (p=0.018; Table 1). Notably, only one (4%) of the 25 programs surveyed utilized social media to connect with potential applicants across both the 2019-2020 and 2020-2021 application cycles, with no change in social media utilization between the application cycles (p>0.05; Table 1).

**Table 1:** Survey questions and responses from a survey administered to orthopedic spine fellowship program directors

Questions with categorical responses	Percentage of program directors
Has your program utilized social media to reach out to applicants in prior years?	
Yes	0%
No	100%
What methods did your program use to inform potential applicants about your program last year? (Mark all that apply)	
Virtual open house/meet-and-greet (zoom or social media)	20%
Mailed materials	16%
Social media	0%
Online videos (i.e. YouTube)	4%
E-mail	56%
Direct fellow or faculty communication	36%
Other	32%
What methods have your program used to inform potential applicants about your program this year, given COVID restrictions? (Mark all that apply)	
Virtual open house/meet-and-greet (zoom or social media)	52%
Mailed materials	12%
Social media	4%
Online Videos (i.e. YouTube)	16%
E-mail	68%
Direct fellow or faculty communication	36%
Other	24%
Has your program added supplemental requirements (additional essays, etc.) for applicants this year compared to previous years?	
Yes	5%
No	95%
How important is the virtual interview this year compared to an in-person interview last year for selecting candidates?	
Significantly less important	5%
Slightly less important	15%
About the same	65%
Slightly more important	5%
Significantly more important	10%
If COVID restrictions were lifted in the future, would you consider virtual interviews as an option in addition to in-person interviews?	
Very unlikely	5%
Unlikely	15%
Undecided	30%
Likely	40%
Very likely	10%
If COVID restrictions were lifted in the future, would you consider virtual interviews to entirely replace in-person interviews?	
Very unlikely	25%
Unlikely	30%
Undecided	35%
Likely	5%
Very likely	5%
Questions with quantitative responses	Mean ± standard deviation
How many orthopedic spine fellowship positions are offered by your program?	2 ± 1
How many applications did your program receive last year?	58 ± 31
How many applications did your program receive this year?	74 ± 30
How many applicants did your program offer interviews to last year?	27 ± 14
How many applicants will your program offer interviews to this year?	41 ± 22
How many separate interviews did each candidate complete during their interview day last year?	4 ± 2
How many separate interviews will each candidate complete during their interview day this year?	4 ± 1
How many interview dates were available last year?	3 ± 5
How many interview dates will be available this year?	4 ± 4

Questions on the general application process

Most programs (95%) did not have supplemental requirements (additional essays, etc.) for applicants in the 2020-2021 cycle relative to the prior year (Table 1). Compared to the prior year, the number of interviews offered by programs increased by 1.5-times (32.7 vs. 21.9 interviews, p=0.024; Table 1). However, there were no differences in the number of applications received by programs, the number of interview dates available, and the number of separate interviews each candidate completed during an interview day (p>0.05 for all; Table 1).

**Figure 1:**
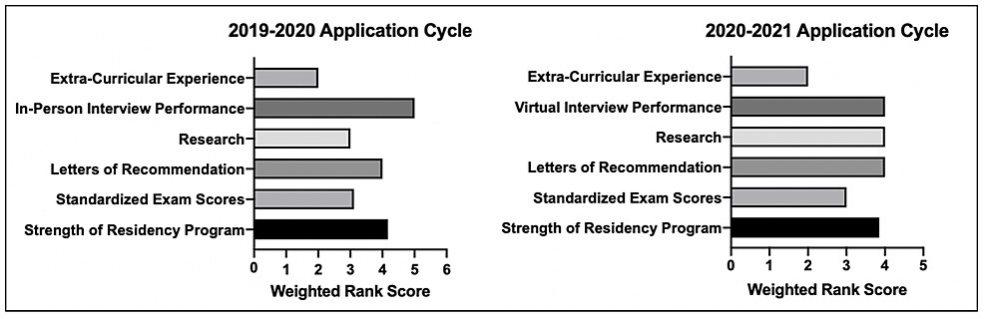
Comparison of the relative importance of various factors for applicant selection in the 2019-2020 vs. 2020-2021 application cycles for orthopedic spine fellowship

In the 2019-2020 application cycle, in-person interview performance was the most important applicant selection factor, as ranked by program directors in our survey (Figure 1). However, in the 2020-2021 cycle which was affected by the pandemic, virtual interview performance, research and letters of recommendation were tied as the most important factors for applicant selection (Figure 1).

Questions on interviews

The majority (65%) of programs thought that virtual interviews in the 2020-2021 cycle were about the same as in-person interviews in the prior year in terms of importance. Half of the respondents (50%) would “likely” or “very likely” consider virtual interviews as an option in addition to in-person interviews if COVID restrictions are lifted in the future. However, most respondents (55%) answered that it was “unlikely” or “very unlikely” that virtual interviews would entirely replace in-person interviews.

## Discussion

Understanding how fellowship application practices have changed in response to COVID-19 is important for continuing improvements in the process. We found that compared to prior years, orthopedic spine fellowship programs more often connected with applicants via virtual open houses during the 2020-2021 cycle, but social media remained nearly unutilized. Programs also offered a significantly greater number of interviews while reweighing candidate metrics. Notably, half of the program directors would consider virtual interviews as a supplement but not a replacement for in-person interviews.

In recent years, fellowship programs across medicine have experimented with virtual platforms to conduct interviews with favorable results [13-15], and COVID-19 appears to have further expedited this trend. To date, one study has discussed the results of utilizing video interviews for orthopedic surgery fellowships [14]. The authors found that both applicants and faculty interviewers for an adult reconstruction fellowship were enthusiastic about the use of video interviews due to the time and costs saved. However, 34% of candidates (n = 52) stated that the video interview had a negative impact on their ranking of the program, and 30% felt that the virtual format was not good for fellowship interviews. Specifically, 19% of candidates were not comfortable ranking the program after a virtual interview, and 15% did not believe that they had the opportunity to present themselves to their satisfaction during the interview [14]. These results demonstrate a consistent controversy in the discussion surrounding the shift to a virtual platform that precludes programs from making the change. However, when surveyed, about 70% of program directors and residents desired changes to be made to the current interview process, as absences for attending interviews have been found to significantly disrupt programs' workflows [10,16]. How fellowship programs have approached the virtual process with universal in-person restrictions during COVID-19 can importantly influence future initiatives for reducing the burden of interviews without sacrificing efficiency.

Compared to the previous year, we found that a greater percentage of spine fellowship programs offered virtual open houses or meet-and-greets to connect with potential applicants during the 2020-2021 application cycle. This finding may illustrate a concrete action taken by programs to combat the known downsides of using a virtual format, as described earlier. Open houses and meet-and-greets provide opportunities for candidates to assess the people, culture, and "feel" of a program [9]. Some of these events include walk-throughs of the institution and city to further recreate the comprehensive experience typically provided by in-person interviews. Adding these events may especially benefit smaller or lesser-known programs or programs in less favorable locations [8], which require stronger recruitment efforts to attract candidates. The experience and comfort gained by programs with a completely virtual application season, especially with hosting such informal events, may help address the concern regarding the ability to gauge fit for applicants with a virtual interview.

Interestingly, spine fellowship programs did not utilize social media as a recruitment tool before or during the pandemic. A study by Wang et al. found that compared to the previous year, orthopedic surgery residency programs saw a 355% increase in social media use during the 2020-2021 application cycle [17]. Similar trends were seen for other specialties, including fellowships for other surgical subspecialties [18-20]. It is unclear why spine fellowship programs have opted to not use social media as a marketing and recruitment tool, as these platforms are effective for providing information to and connecting with potential applicants. Perhaps the required effort for continuous content creation and posting acted as a deterrent for fellowship programs which typically have fewer administrators and total members.

Regarding applicant selection, there was an increased importance placed on letters of recommendation (LOR) and research during the 2020-2021 application cycle relative to the prior year. For the 2019-2020 cycle, these two factors were ranked third and fifth in importance for candidate evaluation, with the in-person interview the single most important factor considered. This aligns with previous reports regarding selection criteria for orthopedic surgery fellowships, with the in-person interview noted to significantly impact applicants' positions on the final rank list [21-23]. However, with the shift to virtual interviews in the 2020-2021 cycle, LOR and research became equally considered in the applicant selection process as the interview. This may represent program directors' doubts about the accuracy of virtual interviews in gauging the professional potential and personal attributes of candidates. Such uncertainty may further be supported by the increase in interview invitations offered this year. Overall, the trends toward a more holistic candidate selection process may represent lasting changes as fellowship programs contemplate greater utilization of virtual platforms.

Finally, we found that while half of the responding program directors would consider virtual interviews as an option in addition to in-person interviews, most answered that it was unlikely that virtual interviews would entirely replace in-person interviews. Considering the doubts surrounding the use of virtual interviews, these findings are in line with our expectations and with the results of previous studies [24]. It is important to emphasize that, on average, orthopedic residents spend 11 days away from training and $5,875 on travel for fellowship interviews [10]. Similarly, host programs incur significant expenses, including events during interview day and lost revenue from canceled clinic appointments and procedures because of faculty participation [25-27]. More than substantial cost savings, interviewing via a virtual format also allows for greater flexibility in scheduling, decreased stress, easier document review, and note-taking during the interview [27]. In addition, offering virtual options may reduce disparities amongst applicants by removing the financial barriers for residents with lower means. More consideration should be given to continuing the use of virtual interviews in the future to lessen the financial burden for applicants and fellowship programs alike.

The results of our study should be interpreted in the context of its limitations. First, there may be response bias amongst the programs that completed our survey, so results may not be generalizable to the entire national sample of spine fellowship programs or fellowship programs for other orthopedic subspecialties. However, our response rate of 33% is on par with prior survey studies administered to orthopedic program directors [21,23,28]. Additionally, the survey was completed by fellowship program directors, and we were unable to analyze the virtual process from an applicant's perspective. Finally, the surveys were administered before match results were released. Therefore, we were unable to examine the satisfaction of programs with their results and how they compare to prior years. Future research should evaluate the success of the 2020-2021 fellowship match process from both program and applicant perspectives to provide evidence for or against the effectiveness of virtual interviews. Studies should also assess whether the virtual application process fostered differences in the diversity of applicants who applied, interviewed, and matched at spine fellowship programs.

## Conclusions

During the 2020-2021 application cycle, spine fellowship programs were more likely to use virtual social events to recruit potential applicants, send out more interview invitations, and equally consider LOR and research with interview performance when evaluating candidates. Further, programs did not utilize social media as a recruitment tool and are unlikely to replace in-person interviews with video interviews. However, half of the program directors would consider offering virtual interviews as an option during future application cycles, which may help reduce costs and barriers for applicants with financial restraints. Fellowship programs should consider the benefits of a virtual application process as a method to decrease expenses and lost clinical time as well as increase diversity in spine surgery.
